# A charismatic new species of green lacewing discovered in Malaysia (Neuroptera, Chrysopidae): the confluence of citizen scientist, online image database and cybertaxonomy

**DOI:** 10.3897/zookeys.214.3220

**Published:** 2012-08-07

**Authors:** Shaun L. Winterton, Hock Ping Guek, Stephen J. Brooks

**Affiliations:** 1 California State Collection of Arthropods, California Department of Food & Agriculture, Sacramento, California, USA; 2 aman Sri Sinar, Kuala Lumpur, Malaysia; 3 Department of Entomology, The Natural History Museum, London, Great Britain

**Keywords:** citizen scientist, cybertaxonomy, Ankylopterygini

## Abstract

An unusual new species of green lacewing (Neuroptera: Chrysopidae: *Semachrysa jade* **sp. n.**) is described from Selangor (Malaysia) as a joint discovery by citizen scientist and professional taxonomists. The incidental nature of this discovery is underscored by the fact that the species was initially photographed and then released, with images subsequently posted to an online image database. It was not until the images in the database were randomly examined by the professional taxonomists that it was determined that the species was in fact new. A subsequent specimen was collected at the same locality and is described herein along with another specimen identified from nearby Sabah.

## Introduction

New species are increasingly being discovered by the general public with interests in the natural sciences long before they are recognized as new to science by professional taxonomists and formally described. With the rapid development of digital photographic technology, professional and amateur photographers are unknowingly discovering and informally documenting new species of animals and plants by placing images of them in online image databases long before taxonomists can examine them. In some cases the specimen is not collected, so this discovery is effervescent until additional specimens can be subsequently vouchered to enable type designation during the formal descriptive process. Herein we document one such case involving a distinctive new green lacewing (Neuroptera: Chrysopidae) species from Malaysia.

Green lacewings are the second largest family of lacewings, with more than 1200 species in approximately 80 valid genera distributed in all major biogeographical regions worldwide (Brooks and Barnard 1990). Three subfamilies are recognized although number of species in the subfamilies Apochrysinae and Nothochrysinae comprise a relatively minor component of the world fauna. Apochrysinae are represented by six pantropical genera of spectacularly large and delicate species (Kimmins 1952; Winterton and Brooks 2002). Nothochrysinae are represented by nine genera world wide, and contain many species that exhibit putative ancestral characteristics (Adams 1967; Brooks and Barnard 1990; Adams and Penny 1992). The overwhelming majority of the generic and species-level diversity belongs to Chrysopinae, with approximately 97% of all living species. This subfamily is further divided into four tribes: Belonopterygini, Chrysopini, Leucochrysini and Ankylopterygini (Brooks and Barnard 1990; Winterton and de Freitas 2006). Ankylopterygini are a Palaeotropical group thoroughly characterized recently by Brooks (1983, 1986) and Brooks and Barnard (1990) and differentiated from other tribes based on the close proximity of veins Sc and R, narrow mandibles lacking internal teeth, and elongation and apical narrowing of the labial and maxillary palpi.  The genus *Semachrysa* Brooks, 1983 contains 14 previously described species from Japan southwards through the Oriental and Australasian regions to Australia (Brooks 1983, 1986). *Semachrysa* is differentiated from other Ankylopterygini genera by the presence of distinct markings (i.e. two or three spots) on the frons, cell *im* present, forewing with an elongate stigma with 3–4 Sc-R crossveins, and male genitalia with two pairs of gonosetae (Brooks and Barnard 1990). A new and distinctive species is described here (*Semachrysa jade* sp. n.) based on two female specimens from Malaysia.

The discovery of a new species of *Semachrysa* described in this paper is a direct result of the incidental interaction of photographer/citizen scientist, online image database and professional scientists. Images of *Semachrysa jade* sp. n. (Figures 2–3) were initially posted by the second author (GHP) on the online image database Flickr ® for comment by the photography and natural history communities. The specimen had been released once it was photographed and at this stage no determination had been made on the taxonomic identity of the species. The online images were then randomly examined by the senior author (SLW) who determined that this distinctive species was not immediately recognizable as any previously described species. Links to the images were forwarded to additional experts in chrysopid taxonomy to elicit comment on its possible taxonomic identity. After extensive discussion it was concluded that the species was likely new to science but its generic placement inconclusive based solely upon the images at hand. The senior author contacted the photographer and an additional specimen to was collected a year later at the same locality (Figure 4). This second specimen was photographed and sent to the senior author for examination and subsequent formal description as a new species of *Semachrysa*. An additional female specimen from Sabah was also located in the entomology collection of the Natural History Museum, London. The manuscript was prepared by the authors based on a cybertaxonomy model using internet cloud based technology (i.e. Google™ Document), while images were archived in Encyclopedia of Life and Morphbank image databases and new taxonomic acts registered in Zoobank (Pyle and Michel 2008).

## Materials and methods

Terminology follows Tjeder (1966) and Brooks and Barnard (1990). High-resolution digital images were deposited into Morphbank with embedded URL links within the document between descriptions and Morphbank images. All new nomenclatural acts and literature are registered in Zoobank (Pyle and Michel 2008). Genitalia were macerated in 10% KOH to remove soft tissue, then rinsed in distilled water and dilute glacial acetic acid, and dissected in 80% ethanol and subsequently stained with a saturated solution of Chlorazol Black in 40% ethanol. Genitalia preparations were placed in glycerine in a genitalia vial mounted on the pin beneath the specimen.

## Taxonomy

### Semachrysa jade sp. n.

urn:lsid:zoobank.org:act:2F358345-BE61-46B5-9672-10C1F9730719

http://species-id.net/wiki/Semachrysa_jade

**Figure 1.**
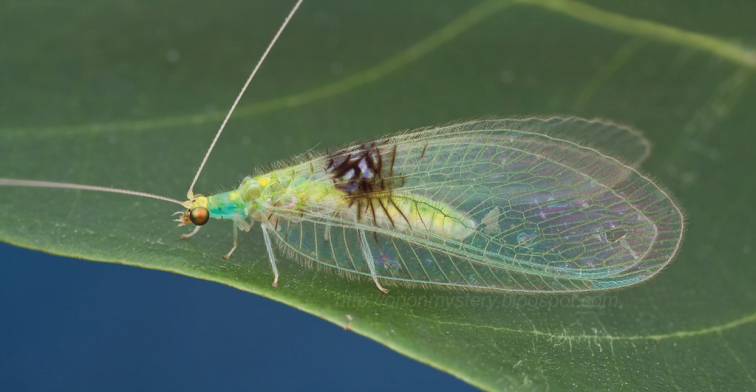
*Semachrysa jade* sp. n., female holotype habitus (Morphbank: 791595). Forewing length: 15.0 mm. Photographer: Guek Hock Ping.

Figures 1 [Fig F2] [Fig F3] [Fig F4] [Fig F5] –6

#### Type material.

**Holotype** female, MALAYSIA: Selangor: Road B57, 0.8 km SSW of entrance of Selangor State Park, (3.3057,101.693) closed forest, Guek Hock-Ping, 27.i.2012 (California Academy of Sciences Collection).

**Paratype:** MALAYSIA: Sabah: Tawau, DR. Lim, 8.vii.1981, on Cocao (Natural History Museum, London).

#### Diagnosis.

Extensive black markings with white fenestrations in basal portion of both wings; forewing with basal four to five crossveins between R and Rs converging posteriorly, Rs closely approximating Psm basally; three crossveins between Cu1 and Cu2, 1st posterior marginal crossvein forked with posterior arm joining to Cu2 petiolate to margin; two dark spots across frons below antennae; single marking between antennal bases; dark markings medially on abdominal tergites 2–4; sternite seven with acuminate posteromedial margin with tuft of short dark setae.

#### Description.

**Figure 2. F2:**
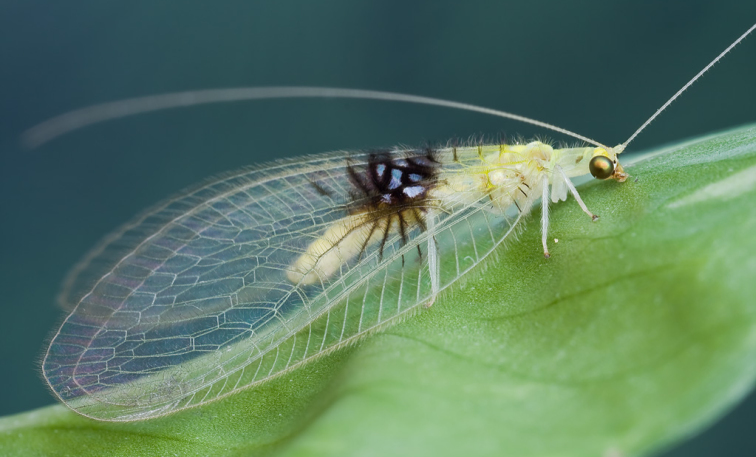
*Semachrysa jade* sp. n. female habitus, specimen that was originally photographed and released (Morphbank: 791596). Forewing length: 15.0 mm. Photographer: Guek Hock Ping.

**Figure 3. F3:**
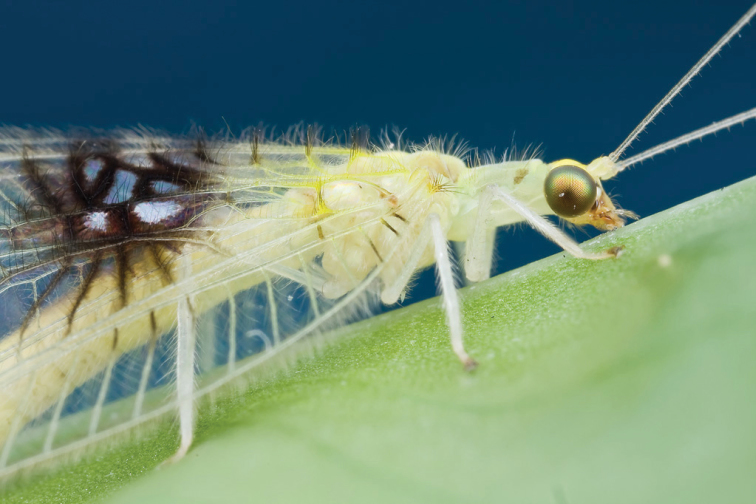
*Semachrysa jade* sp. n. female habitus (Morphbank: 791597). Forewing length: 15.0 mm. Photographer: Guek Hock Ping.

**Figure 4. F4:**
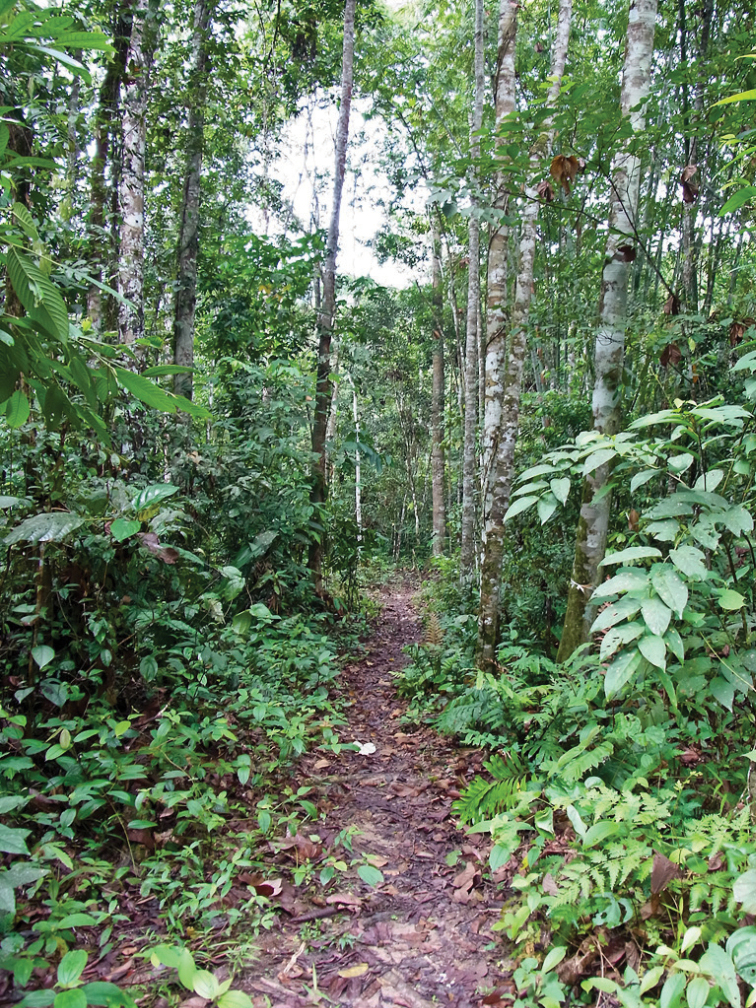
Type locality of *Semachrysa jade* sp. n., closed forest, 0.8 km SSW of entrance of Selangor State Park, Selangor, Malaysia (GPS: 3.3057,101.693). Photographer: Guek Hock Ping.

**Figure 5. F5:**
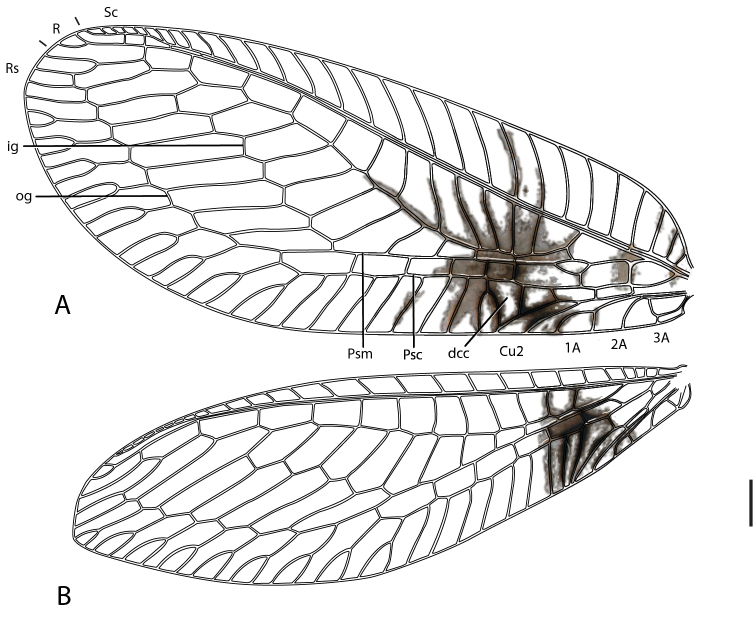
*Semachrysa jade* sp. n. **A** forewing **B** hindwing. Vestiture omitted. Abbreviations: *dcc*, distal cubital cell; *ig*, inner gradate series; *psc*, pseudocubital vein; *psm*, pseudomedial vein; *og*; outer gradate series. Scale line: 1.0 mm.

**Figure 6.**
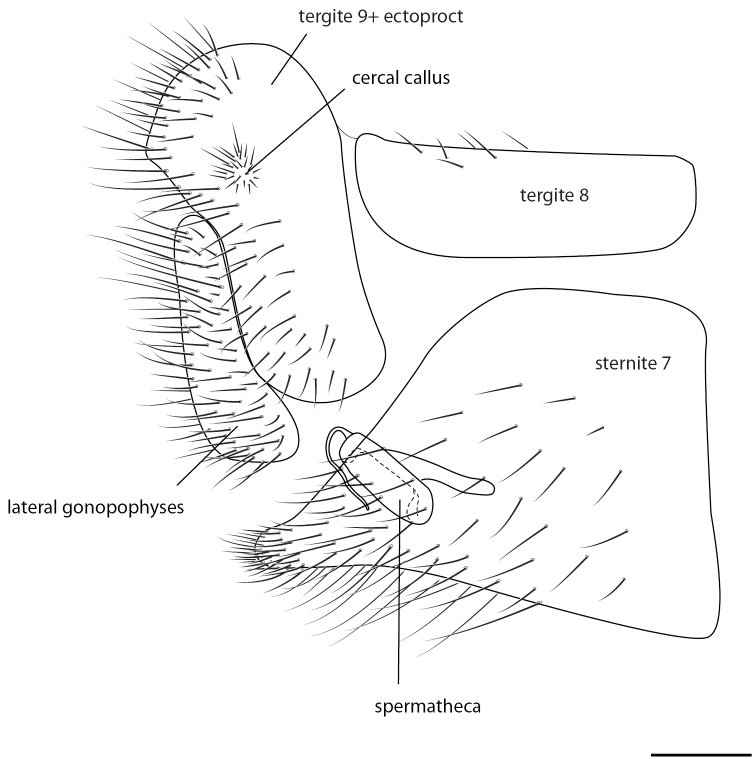
*Semachrysa jade* sp. n. Female terminalia with structures labeled. Scale line: 0.2 mm.

Female: Wing length (forewing: 15.0 mm; hindwing: 13.5–14.0 mm). Overall colouration in live specimens bright green and yellow, with dark markings on head, abdomen and both wings (pale yellow in dried specimens). Head. Yellow with black marking on vertex between antennae and small quadrangular marking on frons below antennal base and proximal to eye margin; clypeus with brown suffusion laterally; labrum emarginate medially; antenna pale green but otherwise unmarked, slightly longer than forewing; flagellum with at least 50 flagellomeres; palpi green, unmarked. Thorax. Prothorax green with small brown mark anterolaterally on pronotum; setae short, green, and relatively sparse, darker and more dense laterally; mesonotum  and metanotum yellow green, scutum darker in preserved specimen, mesoscutellum pale yellow, setae sparse and pale yellow to white; legs very pale green with white setae, setae shorter and yellowish distally on foretibiae; distal tarsomere and claws brownish on all legs; wings relatively rounded, forewing costal area broad, rounded basally, then straight to wing apex; forewing with Rs sigmoid and closely approximating pseudomedial (Psm) basally; first five r-rs crossveins convergent, remaining crossveins sub parallel; seven inner gradate crossveins, meeting Psm; nine outer gradate crossveins; three crossveins between Cu1 and Cu2, 1st posterior marginal crossvein forked with posterior arm joining to Cu2, petiolate to margin; hind wing with five inner gradate crossveins, seven outer gradate crossveins; wing veins with setae relatively elongate and pedicellate, pedicels longer in basal portion of wing, setae colour corresponding to wing markings and colour of wing venation; wing hyaline with markings as per Figure 5, venation mostly pale green, forewing costal crossveins dark anteriorly on crossveins 1–3 and posteriorly on crossveins 8–10; basal subcostal crossvein dark; membrane infuscate and venation dark in medial area of both wings from R vein to posterior margin, markings darker posteriorly with cells (e.g. dcc, c2 and cu2) with white fenestration (Figs 1–4); basal portion of cell m2 infuscate; spot at base of wing on cubital vein; fourth posterior marginal crossvein dark distally. Abdomen. Yellow green dorsally, white ventrally; dark medial stripe on tergites 2–4; poorly defined dark spot laterally on tergites 3–5; sternite 8 with tuft of short strong setae medially. Female terminalia (Figure 6): Trichobothria ca. 25; sternite seven with broadly acuminate posteromedial margin with tuft of short dark setae; subgenitale relatively broad; spermatheca with vela relatively short and straight; ventral impression relatively shallow and wide; duct with slight coil.

#### Comments.

The unusual new species is easily differentiated from all other species of *Semachrysa* by the distinctive wing venation mark between the antennal bases and only two spots across the frons, as well as the female abdominal sternite 7 being posteromedially acuminate with a tuft of strong setae. Only the female is known at this stage. *Semachrysa jade* sp. n. is similar in appearance to *Semachrysa wallacei*, based on head and wing markings. Like *Semachrysa jade* sp. n., *Semachrysa wallacei* is also only known from the female.

#### Etymology.

This new species is named after the daughter of the senior author, Jade Tanya Winterton.

##### Revised dichotomous key to species of *Semachrysa* Brooks

(Modified after Brooks 1983)

**Table:** 

1	Wings unmarked except for indistinct small spot on dcc and black border on costa	*Semachrysa dammermanni* (Esben-Petersen, 1929)
–	Wings with numerous brown or black markings	2
2	Forewing with strong dark brown marking at base of outer gradate series	3
–	Forewing without dark markings in this position (sometimes very faint)	4
3	Large dark brown marking present on fourth posterior marginal crossvein of forewing; small marking present on Cu2; male genitalia with arcessus narrow, without ridges; female genitalia with duct of spermatheca twisted, vela very long	*Semachrysa contorta* Brooks, 1983
–	Small mark present on fourth posterior marginal crossvein of forewing, but with large brown mark on dcc; male genitalia with arcessus broad, ridged; female genitalia with duct of spermatheca and vela short	*Semachrysa matsumurae* (Okamoto, 1914)
4	Single median marking on vertex either between, or immediately posterior to antennal bases	5
–	Medial marking absent on vertex between or immediately posterior to antennal bases	7
5	Extensive dark markings in basal portion of forewing from R vein to posterior margin of wing; basal four to five crossveins between R and Rs converging posteriorly; three crossveins between Cu1 and Cu2, 1st posterior marginal crossvein forked with posterior arm joining to Cu2 petiolate to margin	*Semachrysa jade* sp. n.
–	Dark markings in basal portion of forewing restricted to basal cells along posterior margin of forewing; basal four to five crossveins between R and Rs parallel; two crossveins between Cu1 and Cu2, 1st posterior marginal crossvein not forked and separate from Cu2 to wing margin	6
6	Hindwing with distinct brown marking on dcc and faint shading on posterior marginal crossveins	*Semachrysa wallacei* Brooks, 1983
–	Hindwing with only faint markings on dcc or forked posterior forked marginal crossveins, but marking present on fourth posterior marginal crossvein	*Semachrysa picilabris*(Kimmins, 1952)
7	Prominent markings at base of inner gradate series of forewing and on fourth posterior marginal crossvein; male abdominal setae dense and fine	*Semachrysa papuensis* Brooks, 1983
–	Forewing lacking above combination of markings, though pale suffusion may be present	8
8	Two spots on frons below antennae	*Semachrysa cruciata* (Esben-Petersen, 1928)
–	Three spots on frons below antennae	9
9	Brown band extending from along inner margin of eye from postocular lobe to anterior edge of vertex	*Semachrysapolysticta* Brooks, 1983
–	Brown band absent; isolated mark on postocular lobe	10
10	Fore and hindwing lightly suffused with brown, particularly along posterior margins, distinct spots absent	*Semachrysa minuta* Brooks, 1983
–	Fore and hind light brown suffusion admixed with dark markings	11
11	Darkest marking on forewing on dcc	12
–	Darkest marking on forewing on fourth posterior marginal crossvein	13
12	Forewing dcc with large marking, extending from anal veins to second cubital cell (c2); many crossveins with pale brown suffusion including Rs and inner gradate series	*Semachrysa sagitta* Brooks, 1983
	Forewing dcc with small marking, not extending to anal veins or c2; all crossveins pale	*Semachrysa nigribasis* (Banks, 1920)
13	Small species, forewing length 7.5 mm; four inner gradate and five outer gradate crossveins	*Semachrysa hyndi* Brooks, 1983
–	Larger species, forewing length 9.0 mm or greater; at least six inner gradate and outer gradate crossveins	14
14	Forewing length 9.0 mm, faint marking on dcc with extensive brown suffusion; female spermatheca with deep ventral impression	*Semachrysa decorata*(Esben-Petersen, 1913)
–	Forewing length 13.0 mm, dcc lacking dark markings and brown suffusion posteriorly; female spermatheca with shallow ventral impression	*Semachrysa claggi* (Banks, 1937)

## Supplementary Material

XML Treatment for Semachrysa jade
